# A ‘smart’ tube holder enables real-time sample monitoring in a standard lab centrifuge

**DOI:** 10.1371/journal.pone.0195907

**Published:** 2018-04-16

**Authors:** Tony Hoang, Nicholas Moskwa, Ken Halvorsen

**Affiliations:** 1 The RNA Institute, University at Albany, State University of New York, Albany, New York, United States of America; 2 Department of Chemistry, University at Albany, State University of New York, New York, United States of America; 3 Department of Biology, University at Albany, State University of New York, Albany, New York, United States of America; Public Library of Science, UNITED KINGDOM

## Abstract

The centrifuge is among the oldest and most widely used pieces of laboratory equipment, with significant applications that include clinical diagnostics and biomedical research. A major limitation of laboratory centrifuges is their “black box” nature, limiting sample observation to before and after centrifugation. Thus, optimized protocols require significant trial and error, while unoptimized protocols waste time by centrifuging longer than necessary or material due to incomplete sedimentation. Here, we developed an instrumented centrifuge tube receptacle compatible with several commercial benchtop centrifuges that can provide real-time sample analysis during centrifugation. We demonstrated the system by monitoring cell separations during centrifugation for different spin speeds, concentrations, buffers, cell types, and temperatures. We show that the collected data are valuable for analytical purposes (e.g. quality control), or as feedback to the user or the instrument. For the latter, we verified an adaptation where complete sedimentation turned off the centrifuge and notified the user by a text message. Our system adds new functionality to existing laboratory centrifuges, saving users time and providing useful feedback. This add-on potentially enables new analytical applications for an instrument that has remained largely unchanged for decades.

## Introduction

The centrifuge is used to isolate target components from heterogeneous mixtures by density. In the medical and life sciences, centrifuges can be found in almost every lab and are a vital part of daily lab procedures. Applications for laboratory centrifuges include clinical diagnostics [1], separation of blood components [2], isolation of cells and cellular components [3], parasite identification [4], and purification of macromolecules [5].

One major limitation in using centrifuges for separations is the complex trial and error process needed to determine optimized centrifugation speed and time. This problem is compounded in many biological applications, where over-centrifugation can sometimes cause damage to cells [6]. Unoptimized centrifugation can result in unnecessary over centrifuging (by virtue of waiting longer than needed) or material waste (by virtue of not recovering all of the material). This is well illustrated by several publications that show how well accepted clinical centrifugation protocols are sometimes longer than needed [7–9], lengthening diagnostic procedures and potentially adding to health care costs.

Here we solve these problems with a “smart” centrifuge add-on that enables real-time sample monitoring during centrifugation. Conceptually similar to analytical centrifuges [10,11] and inspired in part by our Centrifuge Force Microscope (CFM) [12–14], we have developed a wireless computerized sensor system in a 3D printed housing that can monitor and transmit sample light absorbance in unmodified commercial centrifuges. We demonstrate use of this smart centrifuge system for monitoring cell separations with a variety of speeds, concentrations, temperatures, and centrifuge brands. The collected data can be used for quality control of samples, as well as to provide active feedback to either the centrifuge or the user. Ultimately, we demonstrate a simple and useful application whereby the centrifuge automatically stops itself and notifies the user (e.g. by text message) upon complete sedimentation of a sample.

## Materials and methods

### Mechanical design and 3D printing

The Smart Tube module was designed to fit within a commercial swinging bucket centrifuge (Sorvall X1R) with 400 mL buckets. Since this is among the smallest commercially available bucket sizes, accommodating other bucket sizes was accomplished with simple mechanical adapters. To the extent possible, we used commercially available parts to facilitate reproduction by other labs. Labeled images, a complete parts list, and files used for 3D printing are given as S1 Fig, S1 Table and S1 File, respectively. The mechanical housing is based around a central receptacle for a 15 mL conical bottom centrifuge tube, positioned on a circular pedestal that makes contact with the bottom of the swinging bucket. Five white light LEDs were spaced perpendicular to the tube with five corresponding light sensors positioned opposite to each LED. A central support around the centrifuge tube holds the light and sensor system, a lithium polymer battery, an onboard computer, and a DC-DC voltage converter. The final mass of the assembly without a centrifuge tube is 128 grams, and the maximum rated load of the bucket is 570 grams. The module is counterbalanced with a 3D printed holder containing a central hole for an opposing centrifuge tube as well as two coin holders on each side of the tube to balance the mass using U.S. quarters (which have a diameter of 24.26 mm, thickness of 1.75 mm, and mass of 5.67 grams). Since both the tube assembly and counterbalance have low centers of mass, we found it sufficient to simply balance the mass using our 3d printed counterbalance.

### Basic electrical and computational design

The electrical system is designed to support the basic functionality of the illumination and light sensing system. The light detectors are photoresistors and the light sources are white LED bulbs. A 3.7v 1000 mAh Lithium-Ion battery is connected to a 5v 1000 mAh step-up converter that supplies 5v (parallel connection) to different boards (Raspberry Pi, sensor board, white LED bulbs, and WIFI) of the module. The Raspberry Pi receives data from the sensor board and sends data to the WIFI board. The sensor board consists of a 3-axis accelerometer (connected to the I2C bus of the Raspberry Pi) and the ADC (analog-to-digital converter) converts the analog signal from the light sensors (with resistors) to a digital signal for the Raspberry Pi (the Raspberry Pi does not have a native analog input so an ADC is required).

The onboard computer boots automatically when the module is powered on, and the system is designed to begin collecting data automatically upon starting the centrifuge. This is accomplished with a connected accelerometer that triggers data collection upon reaching a threshold of 1.1 g. At that point, all 5 detectors collect light intensity data that is temporarily stored in RAM and immediately transferred to an external computer. The onboard computer maintains a 10 second running average to determine when the characteristic plateau has been reached and sedimentation is complete. Source code used in this project is included as supporting information S1 Software.

### Instrument operation

To operate the instrument, the user inserts a 15 mL test tube and turns on the power to the module. Using 2.4ghz WIFI, the module connects to an external computer using SSH (Secure Shell) via a WIFI router with static IP address as a hub. A computer script (see Supplemental Material) is executed on the host computer via SSH to the module. The script then runs on the module to automate the remaining processes without any user intervention. While the module is rotating inside the centrifuge, the running average is calculated from sensor data and obtained running averages are transferred to an external computer via SCP (Secure Copy).

### Auto shutdown and user notification features

To implement the automated shutdown and notification features, we programmed the onboard computer to maintain a 10 second running average from the sensors. The algorithm determined complete sedimentation by comparing values of the running average until they are within a certain threshold (a value of 1 AU in our case). Once this algorithm determined that sedimentation is complete, the module sent a signal (creation of a ‘kill’ file) to the external computer, which ultimately shut down the centrifuge.

The shut down and notification processes were controlled by an additional external computer (Raspberry Pi) equipped with I/O functionality. It is worth noting that this functionality could be replicated without an additional computer by utilizing a computer’s serial port or other I/O hardware. The Raspberry Pi computer continually searched for the presence of the ‘kill’ file on the host computer. Upon finding this file, the Raspberry Pi triggered the centrifuge to stop in one of two different ways. In the first implementation, the computer triggered a remote relay (Etekcity, Inc.) that was inline with the centrifuge’s power source, resetting the centrifuge and cutting power to the rotor to stop spinning. In the second implementation, the computer triggered a servo motor rigged to act as a mechanical button presser to mechanically push the stop button. Simultaneously with either of these two stop functionalities, the user notification by text message was performed using an SMS messaging API (Twilio, Inc.).

### Cell preparation and isolation

SIMS (Immortalised mouse submandibular epithelial cell) cells were cultured in Dulbecco's Modified Eagle Medium/Nutrient Mixture F-12 (DMEM/F12) (Life Technologies, California US) with 10% Fetal Bovine Serum (FBS) (Life Technologies, California US) and 100 μg/mL Penicillin Streptomycin (PS) (Life Technologies, California US) in a 10 cm petri dish in a humidified incubator set to 37°C with 5% CO_2_ in the air atmosphere. Media was aspirated away from SIMS containing plate. The plate was washed with 1X Phosphate Buffered Saline (PBS) (Life Technologies, California US). The cells were enzymatically detached from the plate by incubating cells with 2 mL of 0.25% Trypsin-EDTA (Life Technologies, California US) in a humidified incubator set to 37°C with 5% CO_2_ in the air atmosphere. After enzymatic detachments, the cells were re-suspended in DMEM/F12/10%FBS/PS media. The cells were pelleted by centrifuging at 1500 RPM for 5 minutes. The cell pellet was re-suspended in the testing buffer. Cell concentration was determined by pipetting 10 μL of cell suspension into hemocytometer. The same procedure was followed for NIH3T3. HEK 293FT cells have the same isolation procedure but different growth conditions. 293FT cells were grown using MEM non-essential amino acids (Life Technologies, California US) and 2mM L-Glutamine (Life Technologies, California US) in DMEM/F12/10%FBS/PS media.

### Experimental protocols

Before each experiment, a stock of cells was made and cell concentration determined. The cell stock was suspended in necessary buffer (DMEM without phenol red except where noted).

10 mL of cell stock was placed into a 15 mL conical tube (Crystalgen, Inc.). The conical tube was vortexed at max speed for 10 seconds to insure uniform cell suspension. The tube was carefully seated into the powered-on module such that the conical tubes’ written numerical increments were not blocking the light sensors. The entire module containing the tube was placed into an empty centrifuge bucket within the centrifuge, with the proper counterbalance. The centrifuge temperature was held at 25°C for all runs except for the one run performed at 4°C as noted.

For experiments varying the concentration, SIMS cells were prepared at a high stock concentration and serially diluted 2x with buffer. A single tube of cells was centrifuged in triplicate at 1000 RPM for 5 minutes, vortexing for 10 seconds to resuspend cells between each run. For experiments varying the speed, a single tube of SIMS cells at a fixed concentration of 1.3 x 10^6^ cells/mL was centrifuged in triplicate at 4 different speeds (400 RPM, 600 RPM, 800 RPM, 1000 RPM) for 30 minutes.

For experiments varying buffer type, a stock solution of SIMS cells was separated into three aliquots. Each cell aliquot was centrifuged at 1500 RPM for 5 minutes and re-suspended in one of three buffers; PBS, DMEM without phenol red, and DMEM with phenol red. Each aliquot had a fixed concentration of 1.3 x 10^6^ cells/mL, and each of the three tubes of cells was centrifuged in triplicate at 1000 RPM for 5 minutes. For experiments varying cell types, the different cells were prepared in DMEM without phenol red and each tube of cells was centrifuged in triplicate for 5 minutes at 1000 RPM.

### Data analysis

Since all data were collected in triplicate, analysis was performed on the averaged data. Triplicate curves were averaged and then fit with an exponential curve y = y_0_–A*exp(-kt) (excluding the first 10 seconds which has typically noisy data during startup). Exponential behavior is expected due to a force balance that equates forces proportional to position (centrifugal force and buoyant force) with a force proportional to velocity, or derivative of position (friction force). For graphing purposes, the y-offsets (y_0_) obtained from the exponential fits were subtracted from the data to illustrate how the signal changes depended on concentration. The signal changes at each concentration were determined by the magnitude of A from the exponential fit. The noise level in the experiments was determined by the average standard deviation from the final 100 seconds of the lowest concentration curves. These in particular were chosen because the signal was not expected to change over this region, so our measured signal variation in this region can be interpreted as the noise in the system. Data for speed dependence was processed in the same way, except the data was normalized from 0 to 1 for display purposes. Normalization between 0 and 1 was performed by y_norm_ = [y–(y_0_–A)]/A using y_0_ and A from the fit of the averaged curves. The characteristic sedimentation rates were determined by the values of k from the exponential fits. Data for cell and buffer tests were processed similarly, but the other cell types did not show perfect exponential behavior, so these data were normalized for display by y_norm_ = [y–y_min_]/[y_max_−y_min_].

## Results

**Fig 1:**
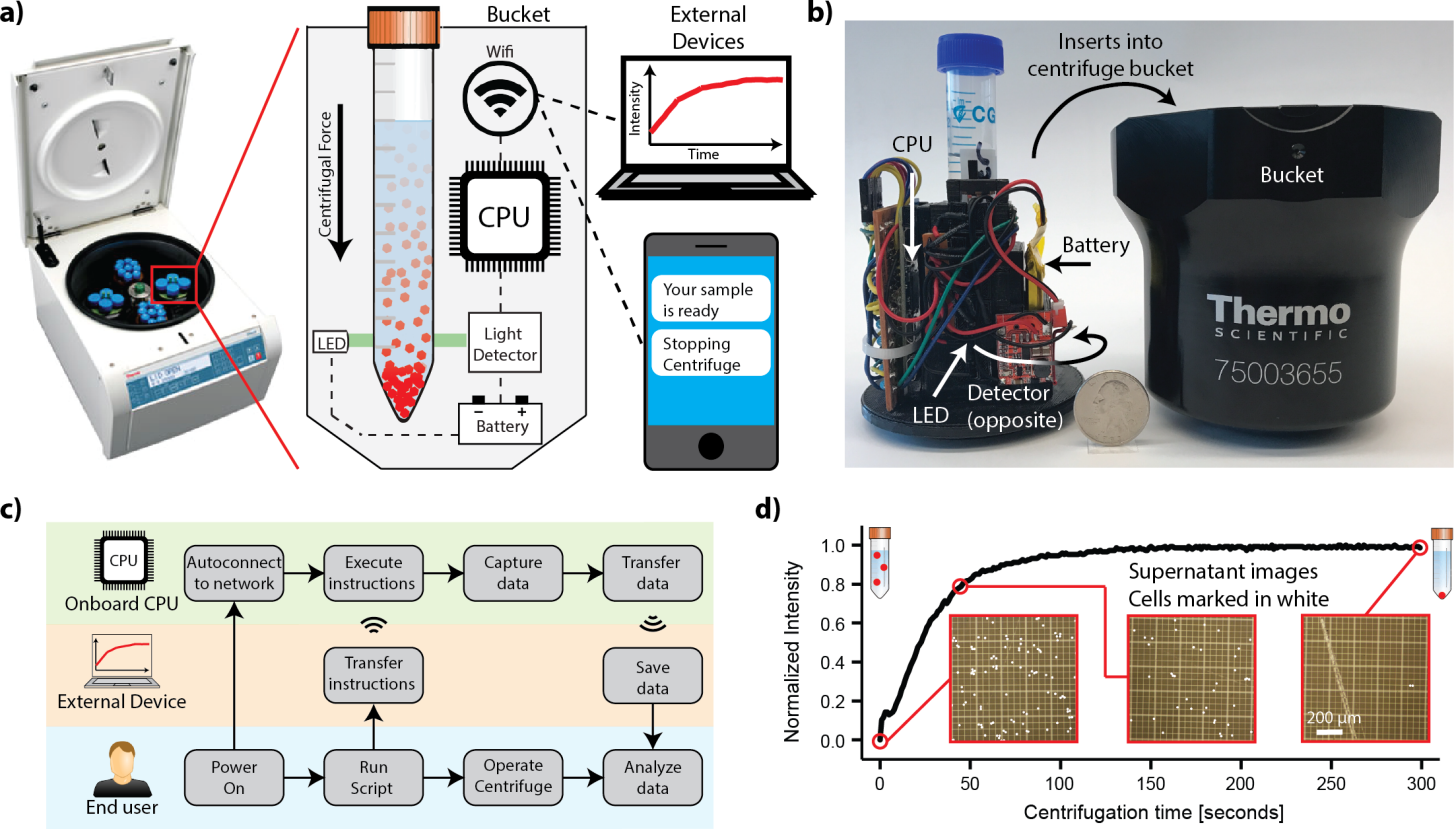
Development and proof of concept. a) Schematic overview and concept for our instrumented centrifuge tube holder, with data sent to external devices, b) Photograph of our prototype device (left) and the standard centrifuge bucket (right) that it is used with, c) A block diagram overview of how the device is used, d) proof of concept data showing an increased light intensity (decreased absorbance) as SIMS cells sedimented at 1000 RPM. Hemocytometer images with cells marked in white show corresponding decrease in cells in the supernatant over time.

Our design goal was to integrate optical absorbance measurements into a commercial benchtop centrifuge without modifying either the centrifuge or the centrifuge tube. To accomplish this goal, we designed an instrumented centrifuge tube holder and a wireless communication system to fit within a standard centrifuge bucket (Fig 1A). We designed the system based on an ordinary commercial swinging-bucket centrifuge (Thermo Scientific Sorvall Legend X1R) with 400 mL buckets (Figs 1B and S1). 3D printing was used to fabricate the mechanical parts of our system, owing to the inexpensive and rapid prototyping ability. 3D printing has become increasingly common for making lab equipment [15–18], and we have used it previously in development of the CFM [13,14]. Using this technique, we developed a housing that fits within the 400 mL swinging buckets and contains a receptacle for a standard 15 mL conical centrifuge tube. A set of five LEDs were positioned to shine light through the sample at different positions, while companion photoresistors detect the transmitted light, forming the basis of the optical system for sample monitoring.

To support the optical system, we additionally housed a computer system (Raspberry Pi Zero) for control, data processing, transmission, and analysis. The computer system was connected to a WIFI adapter, an accelerometer, an electrical system composed of a lithium polymer battery, DC-DC voltage converter, and a power switch. Upon powering up, the onboard computer boots and auto-connects to a pre-assigned wireless network and awaits user commands from a remote desktop (Fig 1C). Once commands are received, the user operates the centrifuge normally, and data collection begins automatically with an accelerometer trigger. The data is transferred wirelessly in real time to the remote desktop for analysis. Alternatively, the computer can be programmed to act on the data in real-time, as we discuss later.

Initial testing showed that a single light source and sensor of the five built into the device was sufficient for monitoring sedimentation, and we chose the pair positioned closest to the conical bottom of the tube. Testing the system under real-world conditions with SIMS cells at 6.5 x 10^6^ cells/mL concentration and spinning at 1000 RPM produced a signal showing exponential behavior (Fig 1D). Taking hemocytometer images of the cell samples taken from the top of the tube at different times confirmed that the signal correlates with sedimentation of the cells (Fig 1D insets).

**Fig 2:**
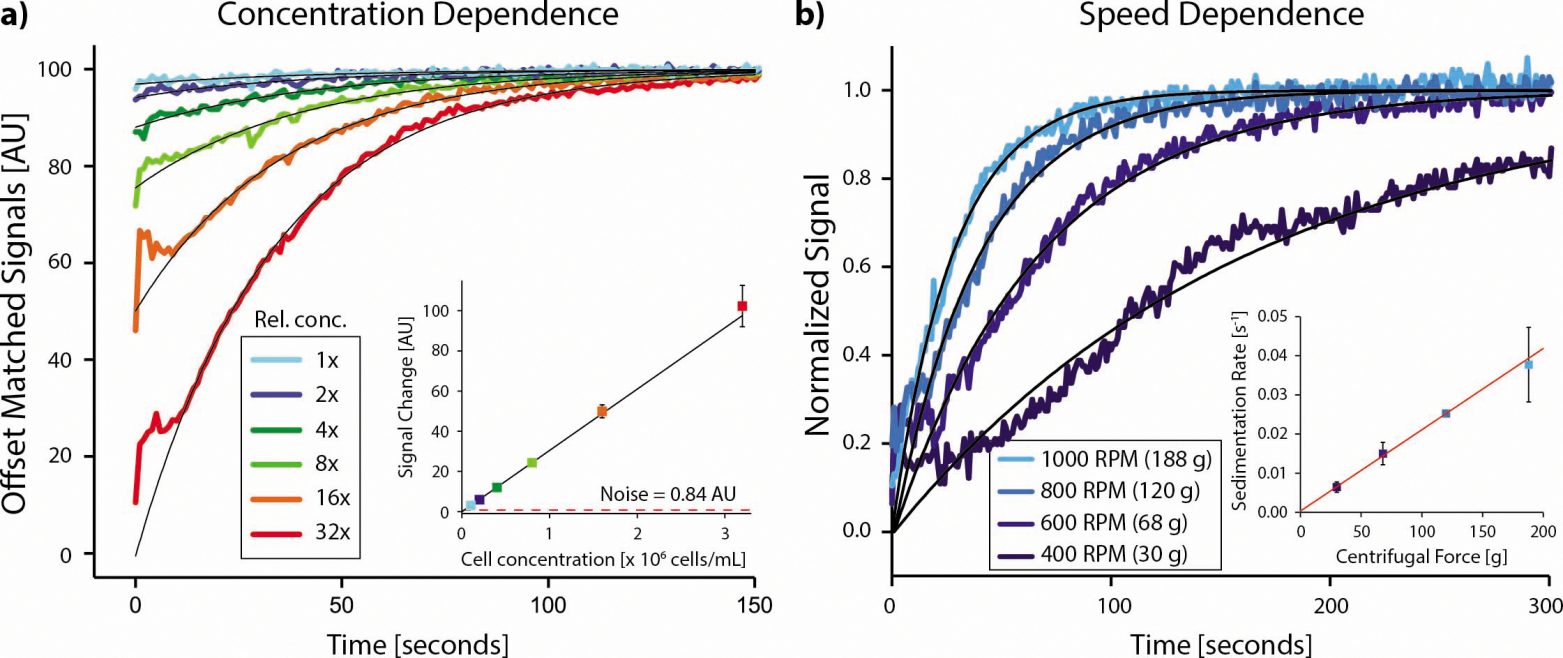
Instrument characterization. a) Data from different cell concentrations (SIMS) show decreased signal at lower concentrations. The magnitude of the signal change is linear with the cell concentration, and the noise level is shown as a dashed line (inset). All data are averaged from triplicates. b) Sedimentation time increases as the speed of centrifugation decreases, as seen visually from normalized sedimentation signals. The sedimentation rate, as determined from exponential fitting to these curves, is approximately linear with centrifugal force as expected (inset). Data points and error bars in the insets represent the mean and standard deviation from the triplicate measurements at each condition.

To determine functional cell concentrations, we performed tests at 1000 RPM for cell concentrations ranging from 3.7 x 10^6^ cells/mL to 0.1 x 10^6^ cells/mL (Fig 2A). As the concentration decreased, the change in signal during centrifugation also decreased as expected while sedimentation rates remained nearly constant. We found a linear relationship between the total signal change (as determined from exponential fits to the data) and the concentration, as would be expected (Fig 2A inset). We were able to monitor cell sedimentation even at our lowest concentration of 0.1 x 10^6^ cells/mL. However, with the current arrangement of light source and detector this is likely close to the detection limit, with a signal to noise ratio ~4.

Next, we varied the centrifuge speed between 400 and 1000 RPM for the same cell type at a concentration of 1.3 x 10^6^ cells/mL. We observed a decreased sedimentation time with increasing centrifugal forces (Fig 2B). Quantitatively, the sedimentation rates determined by exponential fits to the data increased linearly with relative centrifugal force (Fig 2B inset), as is predicted [3].

Having characterized the system and validated its use for a single cell type, we further investigated its use for other biological systems, namely other buffers and cell types. We tested 3 different commonly used buffers: DMEM (Dulbecco’s Modified Eagle Medium) with phenol red, DMEM without phenol red, and PBS (Phosphate Buffered Saline). We tested 3 cell types: an immortalized mouse epithelial cell line (SIMS), a mouse fibroblast cell line (NIH 3T3), and a transformed human embryonic kidney cell line (HEK 293FT). We found in all six conditions that our system tracked the sedimentation, but the different cell types and buffers did affect the apparent sedimentation behavior (S2 Fig).

Building toward practical applications in real laboratory settings, we expanded the utility of the system by demonstrating its use in three different centrifuge models (S3 Fig). Since the 400 mL buckets that we designed for are among the smallest commercially available, adaptation to other centrifuges was accomplished simply by 3D printing structural “adapters” to secure our design in the larger buckets (3D models available in S1 Files). Additionally, since many biological separations are performed below room temperature, we showed that our system could perform equally as well inside the refrigerated centrifuge at 4°C (S4 Fig).

One compelling application of this technology is automation, and to that end we developed and tested software to perform tasks upon complete sedimentation. Using a simple running average algorithm, we were able to automatically determine when sedimentation was complete as the signal plateaued over a 10 second time scale. Upon this software driven event, we programmed the system to send a text message to the user’s phone and stop the centrifuge. We developed two different ways to stop the centrifuge: 1) cut power to a remote relay (see S1 Movie) and 2) send a signal to a prototype mechanical “button presser” that pushed the stop button (see S2 Movie). Both methods were successful in stopping the centrifuge, and commercially available remote button pressers for home automation may make the second method simpler to implement.

## Discussion

Here we have demonstrated proof of concept for an in-centrifuge sample monitoring system that is compatible with both commercial centrifuges and centrifuge tubes. Our prototype system is fully self-contained and fits within a 400 mL centrifuge bucket, so that real time sample monitoring can be achieved with an instrument accessory rather than a new instrument. While such a system could have in principle been developed a decade or more ago, several recent technological advances associated with the “maker movement” have made this work possible. Specifically, the advancement of 3D printing has enabled complex 3D architectures to be rapidly and inexpensively produced. Concurrently, powerful and tiny single board computers such as the Raspberry Pi and shrinking electronics and sensors have enabled powerful analysis, processing, and communication, all of which have been exploited here.

The ability to monitor samples inside a centrifuge comes with many benefits. Perhaps the most obvious ones are saving time and money by easily optimizing centrifuge parameters. Once these parameters are optimized using our monitoring system, they can be readily adapted and used in other centrifuges. Since centrifugation is often a known bottleneck in both research and in clinical diagnostics [19,20], this optimization could have a trickle-down effect to speed research and reduce turnaround time in clinical processing. The potential for automation, as we have shown with ability to notify users or stop the centrifuge upon completion of the run, offers a considerable convenience factor. Furthermore, our system importantly enables collection of data that was previously inaccessible. This data could provide additional quality control during experiments, with information about the cell concentration and even cell type. The data could also lead to new types of experiments that could be performed inside the centrifuge, as it evolves into a more analytical tool.

There are other analytical instruments that utilize centrifugation, most notably the analytical centrifuge [10,11], but also the centrifuge microscope [21,22], and even our own Centrifuge Force Microscope [12–14]. These instruments have some conceptual similarities to the instrument presented here, but they employ different technologies and have different applications that make them functionally quite distinct. The analytical ultracentrifuge is mainly used for obtaining physical and thermodynamic properties of individual or interacting macromolecules. The centrifuge microscope and centrifuge force microscope have mainly been used to observe the effects of physical forces on cells and molecular interactions, respectively. These instruments range in cost from hundreds of thousands of dollars for commercially available analytical ultracentrifuges to a few thousand dollars for custom made centrifuge force microscopes. Unlike these other instruments, our instrument presented here is intended to add analytical functionality to existing preparative centrifugation. Given that the prototype is both inexpensive (material cost of ~$60) and compatible with existing workflows, adding this additional layer of analysis is quite practical.

Considering these benefits, we expect that future work will aim to overcome some of the limitations present in the current prototype. In this work we limited speeds to ~1000 RPM and did not explicitly test higher speeds. Based on previous work with the significantly heavier and more complex CFM operating at similar speeds [12], we believe that it should be possible to accommodate higher speeds, and potentially achieve the top speed of this rotor (5000 RPM) with additional design modifications. A 3D printed housing for an analytical ultracentrifuge has withstood dramatically higher speeds [18]. Most of our electronics are extremely light and small and should withstand higher forces, especially with additional support. In future versions, it would also be useful to make designs accommodating various types and sizes of centrifuge tubes beyond the 15 mL tubes used here, and perhaps expanding to multiple tubes per bucket.

Expanding the range of applications beyond cell separations will present some new challenges. In addition to higher speeds for many applications, volumes can also be significantly smaller. For example, purification of nanoparticles typically use microcentrifuge tubes [23], and blood separations for malaria diagnostics use capillaries [24]. Both of those applications would require significant changes in the optical system, such as employing fiber optics to deliver and retrieve light from the sample with more precision. Such applications may also require redesign of mechanical and electrical components to fit smaller centrifuges that are used for those applications, or to adapt those applications to larger centrifuges.

As our device matures beyond the prototype stage and expands in its applications, safety issues will likely have to be more rigorously addressed. Our prototype withstood a few dozen hours of centrifugation at 1000 RPM with no noticeable problems, but faster speeds, more cycles, and longer durations may stress the system. A key concern is the physical stress on the lithium-ion battery; similar batteries have been known to have safety concerns even under normal operating conditions. More stable battery chemistries may need to be considered. Comprehensive testing of the battery, electronics, and mechanical parts could help to allay fears of putting such an unconventional device inside of a centrifuge.

It is our hope that this work will provide a foundation for a layer of sample analysis that is absent from one of the most common laboratory procedures. Centrifuges have been ubiquitous in the clinic and in the lab for decades, and are seemingly overdue for some technological advancement. Given that we now have the ability to see our samples inside the centrifuge, we see little reason not to exploit it.

## Supporting information

S1 Fig Labeled images of the centrifuge module. (TIF)

S2 Fig Centrifugation with different cell types and buffers. a) Different cell types show different sedimentation behavior, b) Different buffer systems affect both the magnitude of the signal change and the time course of sedimentation.
(TIF)

S3 Fig Demonstration with different centrifuge models. (TIF)

S4 Fig Demonstration at 4°C. (TIF)

S1 Table Bill of materials for the prototype. (PDF)

S1 Files 3D drawing files for all of the printed components. (ZIP)

S1 Software Source code used in this work. (ZIP)

S1 Movie Movie of the system in use with electrical auto stop. (MP4)

S2 Movie Movie of the system in use with mechanical auto stop. (MP4)

S1 Data Raw data used in this work. For each set of experiments, there is one .csv file and one .pdf file describing the conditions. Each experiment has two columns: time (seconds), signal (AU). The data are unprocessed.
(ZIP)
