# Supplementary figures and images for: A ‘smart’ tube holder enables real-time sample monitoring in a standard lab centrifuge
Source: PLoS One. 2018 Apr 16;13(4):e0195907. doi: 10.1371/journal.pone.0195907 (PMC5901991; doi:10.1371/journal.pone.0195907)

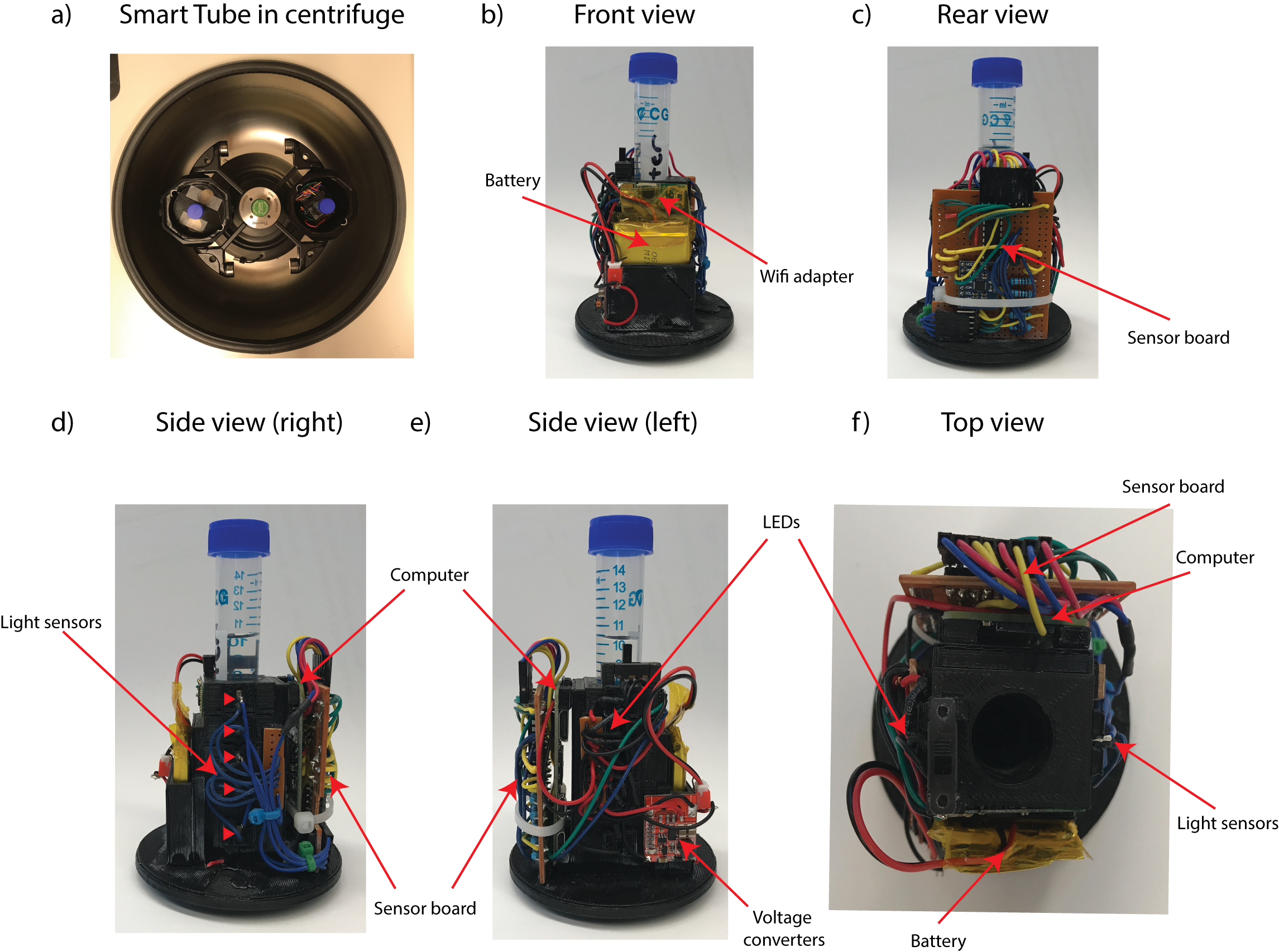

Supplement: S1 Fig — (TIF) [file pone.0195907.s001.tif]

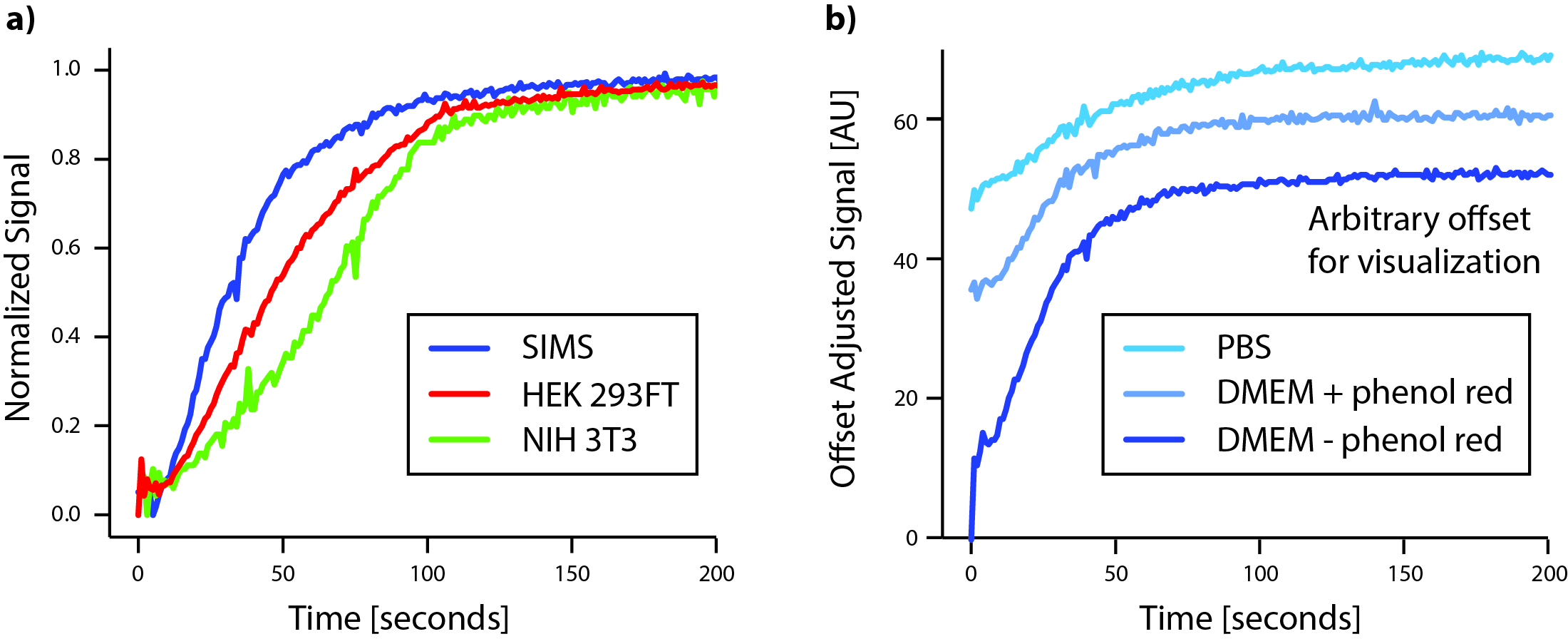

Supplement: S2 Fig — a) Different cell types show different sedimentation behavior, b) Different buffer systems affect both the magnitude of the signal change and the time course of sedimentation. (TIF) [file pone.0195907.s002.tif]

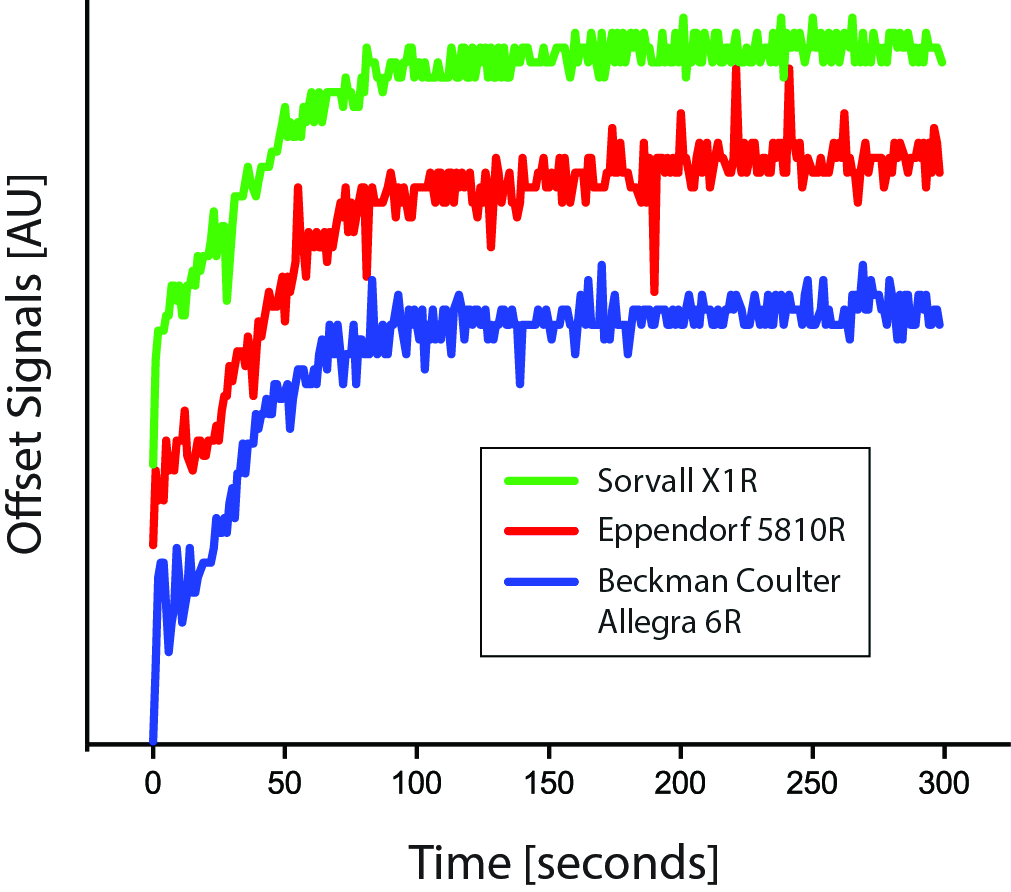

Supplement: S3 Fig — (TIF) [file pone.0195907.s003.tif]

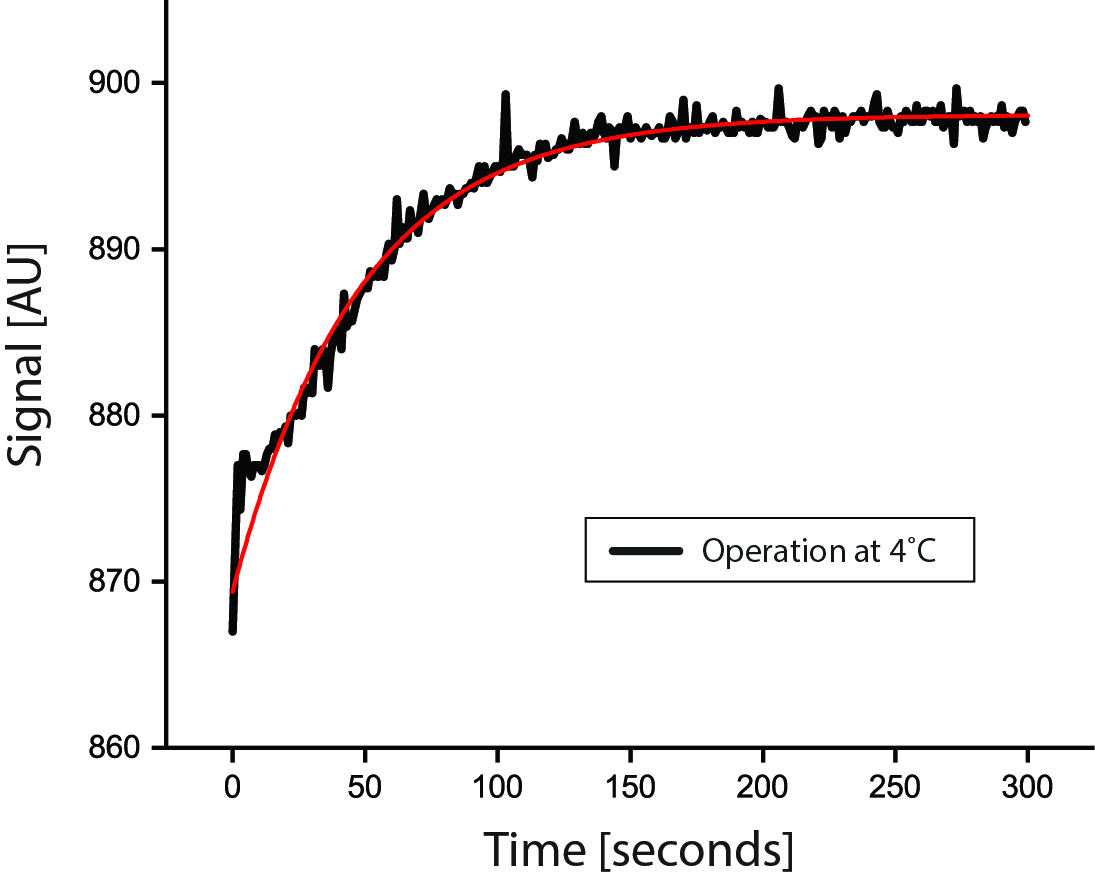

Supplement: S4 Fig — (TIF) [file pone.0195907.s004.tif]
