# Supplementary material for: A ‘smart’ tube holder enables real-time sample monitoring in a standard lab centrifuge
Source: PLoS One. 2018 Apr 16;13(4):e0195907. doi: 10.1371/journal.pone.0195907 (PMC5901991; doi:10.1371/journal.pone.0195907)
Supplement: S1 Table — (PDF) [file pone.0195907.s005.pdf]

ITEM	Manufacturer	Part #	Description	QTY.	Unit Price	Price
1	Raspberry Pi	Zero	SoC microprocessor	1	5.00	5.00
2	CanaKit	CanaKit WiFi Wireless Adapter	USB Wifi - 802.11 n/g/b 150 Mbps	1	9.99	9.99
3	Makerfire	MPU-6050	3 Axis analog gyro sensors 3 Axis Accelerometer Module	1	6.99	6.99
4	Remington	22UL1007STRBLA	22 AWG Gauge Stranded Hook-Up Wire, 300V, 0.0253" Diameter	1	10.35	10.35
5	SUNKEE	GM5539 5539	Photo Light Sensitive Resistor Photoresistor Optoresistor 5mm	5	0.30	1.48
6	Honbay	H&PC-58567	5mm LEDs Pre Wired Light 12V 20cm Bulb	5	0.15	0.74
7	Vktech	4x6cm Double Side PCB	4x6cm Double Side Prototype PCB Universal Printed Circuit Board	1	0.65	0.65
8	Uxcell	1.5mm Pitch Right Angle Male JST	1.5mm Pitch Right Angle Male JST Header Connectors Terminal Red	1	0.02	0.02
9	Uxcell	a13042200ux0676	6 Solder Lug Pin ON/OFF 2 Position Panel Mount Slide Switch	1	0.12	0.12
10	n/a	n/a	3d printed housing	1		0.00
11	DROK	Ultra Small Mini DC Power Module	DC Power Module DC 3V to 5V 1A USB Battery Converter Step Up	1	5.80	5.80
12	Uxcell	10K Ohm, 1/4 Watt, 5%	10K Ohm, 1/4 Watt, 5%, Carbon Film Resistors (pack of 100)	5	0.06	0.29
13	Adafruit	MCP3008	8-Channel 10-Bit ADC With SPI Interface	1	10.81	10.81
14	SparkFun	13813	Lithium Ion Battery - 1Ah	1	9.95	9.95

**Grand Total**	62.18
