# Supplementary material for: A ‘smart’ tube holder enables real-time sample monitoring in a standard lab centrifuge
Source: PLoS One. 2018 Apr 16;13(4):e0195907. doi: 10.1371/journal.pone.0195907 (PMC5901991; doi:10.1371/journal.pone.0195907)
Supplement: S1 Data — For each set of experiments, there is one .csv file and one .pdf file describing the conditions. Each experiment has two columns: time (seconds), signal (AU). The data are unprocessed. (ZIP) [file pone.0195907.s010.zip › S1 Data/cells.pdf]

Run #	Condition	Hemocytometer(cells/mL)
**1**	SIMS	4.57E+06
**2**	SIMS	4.57E+06
**3**	SIMS	4.57E+06
**4**	3T3	1.85E+06
**5**	3T3	1.85E+06
**6**	3T3	1.85E+06
**7**	293FT	5.63E+06
**8**	293FT	5.63E+06
**9**	293FT	5.63E+06

Conditions

**Run Time (minutes)**	5
**RPM**	1000
**Temperature (C)**	25
**Accel**	9
**Decel**	9
**Hemocytometer(cells/mL)**	varied
**Buffer**	DMEM w/o phenyl red
**Cell Type**	varied
**Volume (mL)**	10

*re-suspend cells for 10 sec at max speed on vortex in between runs.
