# Supplementary material for: A ‘smart’ tube holder enables real-time sample monitoring in a standard lab centrifuge
Source: PLoS One. 2018 Apr 16;13(4):e0195907. doi: 10.1371/journal.pone.0195907 (PMC5901991; doi:10.1371/journal.pone.0195907)
Supplement: S1 Data — For each set of experiments, there is one .csv file and one .pdf file describing the conditions. Each experiment has two columns: time (seconds), signal (AU). The data are unprocessed. (ZIP) [file pone.0195907.s010.zip › S1 Data/concentrations.pdf]

Run #	Condition
1	Stock Conc
2	Stock Conc
3	Stock Conc
4	1/2 of Stock Conc
5	1/2 of Stock Conc
6	1/2 of Stock Conc
7	1/4 of Stock Conc
8	1/4 of Stock Conc
9	1/4 of Stock Conc
10	1/8 of Stock Conc
11	1/8 of Stock Conc
12	1/8 of Stock Conc
13	1/16 of Stock Conc
14	1/16 of Stock Conc
15	1/16 of Stock Conc
16	1/32 of Stock Conc
17	1/32 of Stock Conc
18	1/32 of Stock Conc

Conditions

**Run Time (minutes)**	5
**RPM**	1000
**Temperature (C)**	25
**Accel**	9
**Decel**	9
**Hemocytometer of Runs # 10-12 (cells/mL)**	4.6+05
**Buffer**	DMEM w/o phenyl red
**Cell Type**	SIMS

**Volume (mL)**	10

*re-suspend cells for 10 sec at max speed on vortex in between runs.
*Hemocytometer reading only taken at Run # 10-12 because Run # 1-9 are too concentrated.
