# Supplementary material for: A ‘smart’ tube holder enables real-time sample monitoring in a standard lab centrifuge
Source: PLoS One. 2018 Apr 16;13(4):e0195907. doi: 10.1371/journal.pone.0195907 (PMC5901991; doi:10.1371/journal.pone.0195907)
Supplement: S1 Data — For each set of experiments, there is one .csv file and one .pdf file describing the conditions. Each experiment has two columns: time (seconds), signal (AU). The data are unprocessed. (ZIP) [file pone.0195907.s010.zip › S1 Data/speeds.pdf]

Run #	Condition
**1**	1000 RPM
**2**	1000 RPM
**3**	1000 RPM
**4**	800 RPM
**5**	800 RPM
**6**	800 RPM
**7**	600 RPM
**8**	600 RPM
**9**	600 RPM
**10**	400 RPM
**11**	400 RPM
**12**	400 RPM

Conditions

**Run Time (minutes)**	30
**RPM**	varied
**Temperature (C)**	25
**Accel**	9
**Decel**	9
**Initial Hemocytometer (cells/mL)**	1.31E+06
**Buffer**	DMEM w/o phenyl red
**Cell Type**	SIMS
**Volume (mL)**	10

*re-suspend cells for 10 sec at max speed on vortex in between runs.
